# Impact of the COVID-19 lockdown on household diet diversity in rural Bihar, India: a longitudinal survey

**DOI:** 10.1186/s12937-023-00842-z

**Published:** 2023-02-27

**Authors:** Sandra M. Travasso, Smitha Joseph, Sumathi Swaminathan, Anjaly Teresa John, Sanchit Makkar, Patrick Webb, Anura Kurpad, Tinku Thomas

**Affiliations:** 1 Division of Epidemiology and Biostatistics, St. John’s Research Institute, Bengaluru, India; 2 Division of Nutrition, St. John’s Research Institute, Bengaluru, India; 3 Friedman School of Nutrition Science and Policy, Tufts University, Boston, USA; 4 Department of Physiology, St. John’s Medical College, Bengaluru, India; 5 Department of Biostatistics, St. John’s Medical College, 100 Feet Rd, John Nagar, Koramangala, Bengaluru, Karnataka 560034 India

**Keywords:** COVID-19, Lockdown, Longitudinal survey, Diet diversity, India

## Abstract

**Background:**

The COVID-19 pandemic disrupted livelihoods and diets across the world. This study aimed to assess changes in household diet diversity and food consumption between the pre-COVID-19 period (December 2019–January 2020) and during the lockdown (March–May 2020), and to identify the socio-economic characteristics that determine these changes in rural Bihar, India.

**Methods:**

Households that had provided their phone numbers in the pre-COVID-19 household survey (*n* = 1797) were contacted for interviews during the lockdown telephonic survey in a longitudinal survey in two districts (Gaya and Nalanda) in Bihar. In total, 939 households were interviewed. Using data on food consumption from both surveys, 876 households were included in the analysis. Food and Agriculture Organization’s household diet diversity score (HDDS) was used to compare diet diversity between the pre-COVID-19 period and during the lockdown. Logistic regression was used to identify factors affecting household diet diversity and food consumption in the study households.

**Results:**

Low diet diversity increased from 51.6% (95% CI 48.3–54.9) to 75.8% (95% CI 73.0–78.6) from the pre-COVID-19 to the lockdown period. Reduced food consumption was reported across all foods with nearly a quarter of the households reporting reduced consumption of fruits (27%), pulses (25%) and cereals (21%). Nearly 60% and above reported stopping consumption of nutrient-rich foods such as chicken, fish and eggs although the population was predominantly non-vegetarian. Logistic regression analysis revealed that taking a loan from neighbours/relatives (OR = 1.8; 95% CI 1.3–2.5) and belonging to lower social groups (OR = 1.8; 95% CI 1.1–2.9) increased odds of low HDDS. While those possessing ration cards had lower odds of reduced consumption of all food items, it was not associated with stopping consumption of any food item. In an unadjusted analysis, receipt of cash transfer during lockdown was also not associated with diet diversity (OR = 1.2; 95% CI 0.9–1.7).

**Conclusions:**

COVID-19 has impacted the consumption of nutrient-rich foods among already low-income rural households in India. Maintaining diet diversity among socio-economically vulnerable households during periods when food consumption is most threatened by shocks such as COVID-19 would need sustained government support in terms of social protection coverage and benefit transfers in rural communities.

**Supplementary Information:**

The online version contains supplementary material available at 10.1186/s12937-023-00842-z.

## Introduction

The number of people suffering from acute hunger across the world is estimated to have doubled (from the earlier 135 million) as a result of the COVID-19 pandemic [1]. The economic downturn was particularly severe for vulnerable communities in low- and middle-income countries (LMICs) [2]. Emerging literature on the early effects of COVID-19 and child nutrition predict grim outcomes [3–5]. In one estimate, an additional 2.6 million children are predicted to be stunted in 2022 compared to 2019 due to interruptions in nutrition programs and worsening household poverty [3]. Out of this, 30% of the children would be from South Asia [3]. In rural India, the prevalence of stunting and wasting among children below 5 years of age remains high at 37.3 and 19.5% respectively [6]. The condition is worse in some parts of the country like in the state of Bihar, where as many as 44 and 23% of children below 5 years of age are stunted and wasted respectively [7]. These are estimates prior to the onset of COVID-19.

India’s persistently high levels of undernutrition despite high macroeconomic growth has been linked in part to low diet diversity in many rural areas [8–10]. The COVID-19 pandemic compounded that challenge by reducing people’s access to markets, losses in income, and higher prices, thus emphasizing the need for assessment of pandemic impacts which include these dimensions [11]. The Government of India imposed a very stringent lockdown on 25th March 2020 with little warning, and it lasted until 31st May 2020 [12]. Immediate lockdown effects included food market and supply chain disruption [12, 13], curtailed supply and demand for agricultural inputs, labour and commodities, and consumer demand was impacted by loss of income and physical mobility [14]. While the agricultural sector itself recorded positive growth at 3.4% from April–June 2020 (albeit 2.5% point less than the previous quarter), the lockdown represented a significant negative shock [12].

Several studies have shown that the lockdown disproportionately affected the poor, including farmers [11, 12, 15, 16]. To offset the effects of the pandemic, the central government rapidly introduced relief measures in terms of distribution of additional free foodgrains along with other routinely provided food items at subsidized rates through the Public Distribution System [17] and cash transfers [18]. Through our study in Bihar, we observed that households receiving cash transfers were less likely to be food insecure during the pandemic [19]. However, the cash transfers were small [19] while the PDS supplied cereal foodgrains mainly aimed at tackling hunger by meeting energy requirements [20]. Whether these were sufficient measures to ensure diverse diets is not known. It is important to understand the effects of the pandemic and lockdown on household diet diversity as maintaining diet diversity is essential to tackle micronutrient deficiencies [21].

In this context, examining the differences in consumption of various food items before and during the lockdown period contributes to our understanding of impacts on food consumption and diet diversity. Our objective was to examine changes in household diet diversity between the pre-COVID-19 and during the lockdown periods using longitudinal data in rural Bihar state. We also identified the reduction or stoppage in consumption of specific food items during the lockdown. The association of the above with socio-economic characteristics of households, social protection coverage and cash transfers are also examined.

## Methods

### Study setting and participants

Two rounds of household surveys were conducted in Gaya and Nalanda districts of Bihar state, the first between July and August 2019 and the second between December 2019 and January 2020. These surveys examined the household’s own-farm production, distribution, and consumption of five key nutrient-rich foods: namely pulses, milk, egg, chicken and green leafy vegetables (GLVs). We examined government data on district wise production and consumption of nutrient-rich foods to identify two districts in which the study was to be performed. Among all districts, villages in Gaya and Nalanda had high production of nutrient-rich foods, good markets that facilitated trade of agriculture produce in the districts and had no large-scale agriculture/nutrition interventions. The districts were chosen in consultation with government departments of animal husbandry and agriculture. We also sought the advice of researchers working in the area of agriculture in Bihar.

Prior to the first survey, a multistage cluster sampling was used to select 142 and 134 villages in Gaya and Nalanda district, respectively. A Random Walk method [22] was employed to select 10 households per village, to obtain a sample size of 2001 households. Households recruited for the survey were only those with children between 6 months and 60 months of age. To account for heterogeneity in distances to district market centers, villages were situated at 0 to 5 km, 6 to 15 km, 16 to 30 km, and > 30 km distance bands from the district headquarters. Both landholding and landless households that were involved or were not involved in production of nutrient-rich foods such as pulses, GLVs, milk, eggs and chicken were sampled. Broadly, data on socio-economic characteristics of the household, quantity, and cost of consumption of all foods at household level and producer’s information related to production and its cost were collected in this face-to-face survey.

### Data collection for the lockdown survey

A list of all respondents who had shared their phone numbers in the pre-COVID-19 survey was generated for participation in the lockdown survey to collect information about the lockdown period. Interviewers were remotely trained on study objectives and instruments over 5 days over telephonic conference calls. The survey instrument included household details, effect of COVID-19 lockdown on participant income/livelihood, government support, effects on food production and sale (among producers), effects on food purchase and consumption (among all respondents), diet diversity and information on participants general health and hygiene. For the lockdown survey the respondent was the head of the household or a responsible adult household member with knowledge about the household’s food production/livelihood and household food consumption. Data collection was done between August–September 2020. The period of reference for the lockdown survey was March–May 2020 during which period a mandatory lockdown was enforced all over India.

Data collection was undertaken via phone calls which were monitored by listening to randomly selected interview recordings to check for fidelity to the survey instrument. Telephonic conference calls with interviewers were done to discuss any challenges encountered and for giving them feedback on interviews conducted. The telephonic surveys were audio-recorded, and the data was later entered for final analysis.

### Diet diversity and food consumption

To measure household diet diversity, a modified version of the Food and Agriculture Organization’s household diet diversity score was used [23]. Household consumption of eight food items namely cereals, pulses, GLVs, fruits, milk, eggs, fish and chicken were recorded if consumed in the previous 2 months by recall during the pre-COVID-19 survey and during lockdown. The intake of GLVs was specifically focussed upon (but not other vegetables) as it was the nutrient-rich food of interest for our survey. The household diet diversity scores (HDDS) for both surveys were computed as the number of foods reported as consumed in the above list of eight foods. HDDS was classified as low diet diversity if HDDS was less than or equal to median HDDS (HDDS = 6) in the pre-COVID-19 survey. Additionally, since it was not possible to obtain quantitative details of consumption of the foods through the telephonic survey, we collected responses on whether they had reduced or stopped the consumption of each of these food groups as compared to the pre-COVID-19 period, provided they reported consuming the food before the pandemic.

### Household characteristics

Information on household characteristics was collected in the pre-COVID-19 survey. This included social group (scheduled caste/scheduled tribe/others, other backward classes, and forward caste), type of land owned (no land/homestead only/homestead and other land, wherein, land ownership implies ownership of farm-land while homestead is a piece of land on which there is a house), principal occupation (self-employed-agriculture/self-employed-non-agriculture/regular wage or salary earner/casual laborer), type of diet consumed (vegetarian/non-vegetarian) proof of participation in social safety net programs such as possession of Mahatma Gandhi National Rural Employment Guarantee Act 2005 (MGNREGA, an Indian labour law guaranteeing minimum employment in rural areas with at least 100 days of work per year with an average wage of ~USD 2.37 per person per day) [24] card (yes/no), possession of ration (Public distribution system or PDS) card (yes/no) and households with children (1–16 years of age) benefitting from government supplementary nutrition programs (yes/no). The supplementary nutrition programs are meant to provide one meal to the child and include foods such as rice, pulses and boiled egg/sprouted gram distributed through the Integrated Child Development Scheme for 6 months to 6 year old children [25, 26] and Mid-day meal scheme including grains, pulses and vegetables provided at schools [27].

Total household expenditure was calculated, and the monthly per capita consumer expenditure (MPCE) on the specific food items was determined separately. Quintiles of MPCE, which was calculated by dividing total household expenses by household size, were constructed for the analysis.

Specific questions to understand the effect of the lockdown such as “took loan from neighbours/relatives (Yes/No), household in which child feeding was affected with government supplementary nutrition program closure (Yes/No) and small cash transfers (~6.67 USD per month) by the government during the lockdown” [18] were obtained in the lockdown survey.

### Statistical analysis

Standard descriptive statistics are presented as frequency and percentages. The HDDS pre-COVID-19 and during lockdown period were compared using Wilcoxon signed rank test. A series of multiple logistic regression models were used to estimate the odds ratios for low HDDS, reduced consumption and stopped consumption of each food namely cereals, pulses, GLVs, fruits, milk, eggs, fish and chicken. Other than principal occupation (colinear with land ownership) and type of diet (very low proportion of reported vegetarians) all variables were considered in the logistic regression including household characteristics, MPCE, took loan from neighbours/relatives and households in which child feeding was affected with government supplementary nutrition program closure. Analyses were performed on data from households which had data for both surveys. Statistical analysis was performed using SPSS software version 25.

**Fig. 1:**
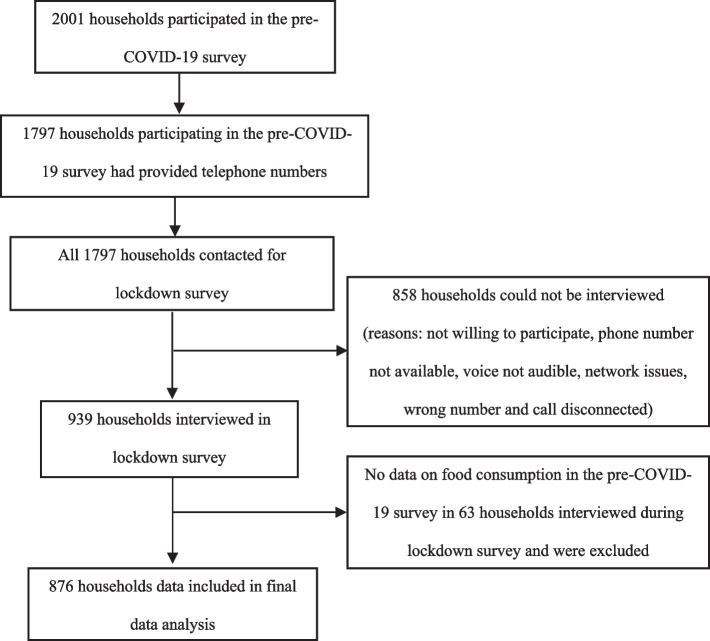
Flowchart for selection of households included in lockdown telephonic survey and data analysis

Multiple attempts were made to contact the pre-COVID-19 survey participants over telephone during the lockdown survey and the final response rate was 52.3%. Thus, a total of 939 households were interviewed telephonically (Fig. 1). Data from 63 households that did not have data on food consumption in the pre-COVID-19 survey were excluded for this analysis, resulting in a final sample size of 876 households. The main analysis presented in the paper includes pre- and post- data analysis of those who participated in the lockdown survey.

## Results

### Household characteristics

Table 1 indicates the characteristics of the households participating in the COVID-19 lockdown survey (*N* = 876) and those not participating in the COVID-19 lockdown survey (*N* = 775). The households participating and those not participating in the lockdown survey were comparable in their characteristics. Data on household characteristics was unavailable for the remaining 146 households. The median household size increased by 1 during lockdown (6 in pre-COVID-19 to 7 in lockdown survey). The principal occupation of most households was agriculture, with 46% of them being self-employed in agriculture. Roughly a quarter of household income was from casual labour work and approximately 30% self-employed in non-agriculture activities or as regular wage/salary earner. About 54% of households procured rations from the public distribution system, 11% availed the guaranteed “right to employment” (MGNREGA) scheme and 35% households had children benefitting from government supplementary nutrition programs. There were few households reporting no land ownership (4.3%) and they were clubbed with the homestead only category for further analysis.

**Table 1:** Background characteristics of participating and non-participating households based on data from the pre-COVID-19 survey

Background characteristics	Participating in COVID-19 lockdown survey (*N* = 876) n (%)	Not participating in COVID-19 lockdown survey (*N* = 775) n (%)
Principal Occupation		
*Self-employed (Agriculture)*	399 (45.6)	352 (45.4)
*Self-employed (Non-Agriculture)*^a^	102 (11.6)	76 (9.8)
*Regular wage/salary earner*	163 (18.6)	156 (20.1)
*Casual laborer*	212 (24.2)	191 (24.7)
MPCE^b^		
*< =1247.4*	176 (20.1)	172 (22.2)
*1247.5–1674.5*	175 (20.0)	154 (19.9)
*1674.6–2150.1*	174 (19.8)	156 (20.1)
*2150.2–2947.0*	176 (20.1)	143 (18.5)
*> 2947.0*	175 (20.0)	149 (19.3)
Social group^b^		
*SC/ST*	251 (29.2)	247 (32.8)
*OBC*	503 (58.5)	422 (55.9)
*Forward castes*	106 (12.3)	85 (11.3)
Religion		
*Hinduism*	851 (97.1)	741 (95.6)
*Islam*	25 (2.9)	34 (4.4)
Type of family		
*Nuclear*	306 (34.9)	291 (37.5)
*Joint/Extended*	570 (65.1)	484 (62.5)
Type of land owned		
*No land*	38 (4.3)	26 (3.3)
*Homestead only*	345 (39.4)	285 (36.8)
*Homestead and other land*	493 (56.3)	464 (59.9)
Possess MGNREGA job card		
*Yes*	98 (11.2)	69 (8.9)
*No*	778 (88.8)	706 (91.1)
Possess ration card		
*Yes*	474 (54.1)	410 (52.9)
*No*	402 (45.9)	365 (47.1)
Type of diet		
*Vegetarian*	41 (4.7)	41 (5.3)
*Non-vegetarian*	835 (95.3)	734 (94.7)
Households with children benefitting from government supplementary nutrition programs^b^		
*Yes*	304 (34.7)	254 (32.8)
*No*	572 (65.3)	521 (67.2)
Households receiving cash transfer from government schemes during lockdown^c^		
*Yes*	369 (42.1)	–
*No*	507 (57.9)	–

*MPCE* Monthly per capita expenditure, *SC/ST* Schedule castes/Schedule tribes, *OBC* Other backward classes, *MGNREGA* Mahatma Gandhi National Rural Employment Guarantee Act

^a^Self-employed (Non-Agriculture) includes shop-keepers, cart-sellers, rickshaw pullers, etc

^b^Social group (*n* = 860) and households with children benefitting from government supplementary nutrition programs (*n* = 860) had 16 missing cases in the COVID-19 lockdown survey group and MPCE (*n* = 774) had 1 missing case and social group (*n* = 754) had 21 missing cases on the group not participating in the COVID-19 lockdown survey

^c^From lockdown survey

### Household diet diversity

Table 2 shows that the prevalence of low HDDS (≤6) was similar in the households participating in the pre-COVID-19 survey (54.0%) and those participating in the lockdown survey (51.6%). Therefore, the households participating in the lockdown survey can be considered to be representative of the population in the pre-COVID-19 survey. The prevalence of low HDDS in the sub-sample from the pre-COVID-19 survey increased significantly from 51.6% to 75.8% during the lockdown (*p* < 0.001).

**Table 2:** Prevalence rates of low HDDS (≤6)

	Pre-COVID-19 survey households (*N* = 1651)	Pre-COVID-19 survey subsample of households followed up in lockdown survey (*N* = 876)	Lockdown survey (*N* = 876)
Prevalence rate of low HDDS (95% CI)	54.0 (51.6–56.4)	51.6 (48.3–54.9)	75.8 (73.0–78.6)

*CI* Confidence interval

The HDDS decreased from a median of six (Q1 = 5, Q3 = 7) pre-COVID-19 to five (Q1 = 3, Q3 = 6) out of eight food groups, during the lockdown period (p < 0.001). In an unadjusted logistic regression analysis, households in the lowest income quintile (MPCE), those belonging to the SC/ST (Schedule Castes/Schedule Tribes) and OBC (Other Backward Classes) social groups, and those that took loan from neighbours/relatives were all at higher odds of low HDDS (Table 3) during the lockdown period. After adjustment for confounders, OBC households and those who had taken loan from neighbours/relatives were at 80% higher odds of low HDDS, as compared to the forward caste and those who had not taken a loan from neighbours/relatives AOR = 1.8; 95% CI 1.1–2.9 and AOR = 1.8; 95% CI 1.3–2.5 respectively. Receipt of cash transfer during lockdown was not associated with diet diversity (OR = 1.2; 95% CI 0.9–1.7).

**Table 3:** Unadjusted and adjusted odds ratios for factors affecting low household diet diversity during lockdown period^a^

Household characteristics		Low household diet diversity score (categorized as ≤6)		
		N	OR (95%CI)	AOR (95%CI)
MPCE	*<=1247.4*	168	1.6 (1.0–2.7)	1.4 (0.8–2.4)
	*1247.5–1674.5*	174	1.2 (0.7–1.9)	1.1 (0.6–1.8)
	*1674.6–2150.1*	173	1.2 (0.7–1.9)	1.1 (0.6–1.8)
	*2150.2–2947.0*	171	1.0 (0.6–1.7)	1.1 (0.6–1.8)
	*> 2947.0*	174	1	1
Social group	*SC/ST*	251	1.8 (1.1–2.9)	1.6 (0.9–2.8)
	*OBC*	503	1.9 (1.2–3.0)	1.8 (1.1–2.9)
	*Forward castes*	106	1	1
Type of land owned	*Homestead only*	374	1.1 (0.8–1.5)	1.0 (0.7–1.4)
	*Homestead and other land*	486	1	1
Household possess MGNREGA job card	*Yes*	98	1.1 (0.7–1.9)	1.1 (0.6–1.8)
	*No*	762	1	1
Household possess ration card	*No*	395	0.9 (0.6–1.2)	1.1 (0.8–1.5)
	*Yes*	465	1	1
Household’s child feeding affected with government supplementary nutrition program closure	*Yes*	155	1.3 (0.8–2.0)	1.2 (0.7–1.9)
	*No*	705	1	1
Took loan /borrow from neighbours/relatives	*Yes*	562	1.9 (1.4–2.6)	1.8 (1.3–2.5)
	*No*	298	1	1

*OR* Odds ratio, *CI* Confidence interval, *AOR* Adjusted odds ratio, *MPCE* Monthly per capita expenditure, *SC/ST* Schedule castes/schedule tribes, *OBC* Other backward classes, *MGNREGA* Mahatma Gandhi National Rural Employment Guarantee Act

^a^Analysis using logistic regression

### Changes in food consumption

**Fig. 2:**
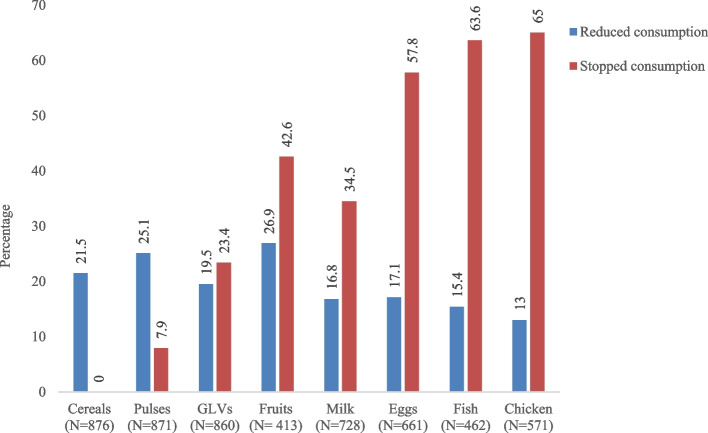
Changes in food consumption among households during the lockdown period

Figure 2 presents data on reduced or stopped consumption of the 8 different foods by households, during lockdown compared to the pre-COVID-19 period. For a given food, lesser proportion reported reduced consumption, and a larger proportion of households reported stopping consumption except in the case of cereals and pulses wherein a higher proportion had reduced consumption rather than stopped consumption. Reduction in food consumption was reported across all foods with nearly a quarter of the households reporting reduced consumption of fruits (27%), followed by pulses (25%) and cereals (21%). As many as 60% and above reported stopping consumption of chicken, fish and eggs although the population was predominantly non-vegetarian.

### Factors associated with changes in food consumption

To assess the factors associated with reduced consumption of foods, a multiple logistic regression was conducted (Table 4). Households belonging to SC/ST social class were more likely to have reduced consumption of cereals (AOR = 3.8; 95% CI 1.8–8.0) and milk (AOR = 2.8; 95% CI 1.2–6.6) as compared to the forward caste. Households that owned a homestead alone had higher odds for reduced consumption of fruits, GLVs and pulses compared to those who owned homestead and other land. Households possessing MGNREGA job card had higher odds for reduced consumption of fish, chicken and fruits while those possessing ration cards had lower odds of reduced consumption of all food items. Households that took loan from neighbours/relatives had higher odds for reduced consumption of cereals (AOR = 1.6; 95% CI 1.1–2.4), pulses (AOR = 2.5; 95% CI 1.7–3.7), and milk (AOR = 1.8; 95% CI 1.1–2.9), as it is likely that financial crisis during the period prompted them to take loan but these loans were insufficient to meet diet diversity and most probably used for other purposes.

**Table 4:** Adjusted odds ratios for household characteristics associated with reducing consumption of foods^a^

	Cereals		Pulses		GLVs		Fruits		Milk		Egg		Fish		Chicken	
	N	AOR (95%CI)	N	AOR (95%CI)	N	AOR (95%CI)	N	AOR (95%CI)	N	AOR (95%CI)	N	AOR (95%CI)	N	AOR (95%CI)	N	AOR (95%CI)
MPCE																
*< =1247.4*	168	0.9 (0.5–1.6)	153	0.8 (0.5–1.5)	124	0.8 (0.4–1.4)	34	0.6 (0.2–1.8)	78	1.6 (0.8–3.1)	53	0.9 (0.4–2.0)	24	1.8 (0.5–6.0)	32	4.5 (1.4–14.1)
*1247.5–1674.5*	174	1.4 (0.8–2.5)	158	1.3 (0.7–2.2)	120	0.5 (0.3–1.0)	37	0.4 (0.1–1.1)	83	1.1 (0.5–2.3)	51	0.7 (0.3–1.6)	39	1.1 (0.3–3.2)	42	2.8(1.0–7.9)
*1674.6–2150.1*	173	1.4 (0.8–2.5)	158	1.2 (0.7–2.1)	139	0.8 (0.4–1.4)	48	1.1 (0.4–2.8)	100	0.9 (0.4–1.9)	57	1.1 (0.5–2.4)	27	1.7 (0.5–5.4)	40	1.9 (0.6–5.4)
*2150.2–2947.0*	171	0.9 (0.5–1.7)	160	1.0 (0.6–1.7)	125	1.1 (0.6–2.0)	44	0.9 (0.3–2.4)	96	1.1 (0.6–2.2)	53	1.4 (0.6–3.2)	41	3.0 (1.0–8.6)	44	1.3 (0.4–3.7)
*> 2947.0*	174	1	159	1	135	1	49	1	115	1	62	1	36	1	39	1
Social group																
*SC/ST*	251	3.8 (1.8–8.0)	230	1.4 (0.7–2.7)	178	1.9 (0.9–3.9)	56	0.7 (0.2–1.9)	110	2.8 (1.2–6.6)	84	1.2 (0.5–3.1)	58	2.3 (0.7–7.2)	58	0.4 (0.1–1.2)
*OBC*	503	2.4 (1.2–4.9)	459	1.6 (0.9–2.8)	379	1.9 (1.0–3.8)	114	1.5 (0.7–3.4)	293	2.0 (0.9–4.3)	154	1.6 (0.7–3.7)	84	2.1 (0.7–6.1)	109	0.4 (0.2–1.1)
*Forward castes*	106	1	99	1	86	1	42	1	69	1	38	1	25	1	30	1
Type of land owned																
*Homestead only*	374	0.6 (0.4–0.8)	353	1.4 (1.0–2.0)	271	1.8 (1.2–2.7)	78	2.8 (1.4–5.7)	167	1.5 (0.9–2.4)	116	1.4 (0.8–2.5)	71	1.0 (0.4–2.1)	85	0.8 (0.4–1.7)
*Homestead and other land*	486	1	435	1	372	1	134	1	305	1	160	1	96	1	112	1
Household possess MGNREGA job card																
*Yes*	98	1.2 (0.7–2.0)	92	1.2 (0.7–1.9)	73	1.2 (0.6–2.1)	23	4.7 (1.6–13.9)	43	1.0 (0.4–2.0)	32	2.0 (0.9–4.5)	16	4.2 (1.1–15.1)	16	5.1 (1.5–17.6)
*No*	762	1	696	1	570	1	189	1	429	1	244	1	151	1	181	1
Household possess ration card																
*No*	395	0.9 (0.6–1.3)	359	1.0 (0.7–1.4)	300	0.8 (0.5–1.2)	110	0.9 (0.5–1.7)	220	1.1 (0.7–1.7)	127	0.7 (0.4–1.3)	76	0.3 (0.1–0.7)	87	0.5 (0.2–0.9)
*Yes*	465	1	429	1	343	1	102	1	252	1	149	1	91	1	110	1
Household’s child feeding affected with government supplementary nutrition program closure																
*Yes*	155	1.5 (1.0–2.2)	136	1.0 (0.6–1.5)	105	1.5 (0.9–2.4)	44	2.5 (1.1–5.5)	70	1.4 (0.7–2.4)	47	1.0 (0.5–2.0)	30	1.4 (0.5–3.5)	38	0.9 (0.4–2.2)
*No*	705	1	652	1	538	1	168	1	402	1	229	1	137	1	159	1
Took loan /borrow from neighbours/relatives																
*Yes*	562	1.6 (1.1–2.4)	510	2.5 (1.7–3.7)	408	1.5 (1.0–2.3)	117	1.7 (0.9–3.2)	280	1.8 (1.1–2.9)	158	1.5 (0.9–2.5)	97	1.7 (0.8–3.6)	109	1.4 (0.7–2.8)
*No*	298	1	278	1	235	1	95	1	192	1	118	1	70	1	88	1

*AOR* Adjusted odds ratio, *CI* Confidence interval, *GLVs* Green leafy vegetables, *MPCE* Monthly per capita expenditure, *SC/ST* Schedule castes/schedule tribes, *OBC* Other backward classes, *MGNREGA* Mahatma Gandhi National Rural Employment Guarantee Act

^a^Analysis using logistic regression

Univariate analysis for factors affecting reduction in consumption of food items is given in a table (see Additional file 1).

The results for multiple logistic regression for cutting certain foods from the diet are presented in Table 5. Households that took loan from neighbours/relatives had higher odds for stopping consumption of chicken (AOR = 1.7; 95% CI 1.1–2.4), milk (AOR = 1.6; 95% CI 1.1–2.2) and eggs (AOR = 1.5; 95% CI 1.1–2.1). Compared to households from the forward class social group, those belonging to the Other Backward Class social group were nearly twice as likely to stop consumption of fish (AOR = 2.5; 95% CI 1.2–4.9), eggs (AOR = 2.1; 95% CI 1.2–3.7) and chicken (AOR = 1.9; 95% CI 1.0–3.3). Households’ possession of ration card was not associated with stopping of any food consumption.

**Table 5:** Adjusted odds ratios for household characteristics associated with stopping consumption of foods^a^

	Pulses		GLVs		Fruits		Milk		Egg		Fish		Chicken	
	N	AOR (95%CI)	N	AOR (95%CI)	N	AOR (95%CI)	N	AOR (95%CI)	N	AOR (95%CI)	N	AOR (95%CI)	N	AOR (95%CI)
MPCE														
*< =1247.4*	165	1.0 (0.4–2.3)	166	1.2 (0.7–2.1)	73	1.0 (0.5–1.9)	129	1.5 (0.9–2.6)	125	1.0 (0.6–1.6)	79	1.1 (0.6–2.2)	110	1.0 (0.6–1.9)
*1247.5–1674.5*	173	1.2 (0.5–2.6)	168	1.4 (0.8–2.5)	72	0.8 (0.4–1.5)	139	1.5 (0.9–2.6)	134	1.1 (0.7–1.9)	99	0.8 (0.4–1.4)	113	0.7 (0.4–1.3)
*1674.6–2150.1*	173	1.0 (0.4–2.2)	170	0.7 (0.4–1.3)	82	0.6 (0.3–1.2)	141	1.0 (0.6–1.7)	132	1.0 (0.6–1.7)	90	1.3 (0.7–2.5)	111	0.8 (0.4–1.5)
*2150.2–2947.0*	171	0.8 (0.3–1.8)	164	1.2 (0.7–2.0)	81	0.8 (0.4–1.5)	149	1.4 (0.8–2.3)	123	1.0 (0.6–1.7)	89	0.6 (0.3–1.2)	115	0.7 (0.4–1.3)
*> 2947.0*	173	1	169	1	94	1	158	1	135	1	94	1	112	1
Social group														
*SC/ST*	249	1.5 (0.5–3.9)	237	1.3 (0.7–2.5)	113	1.5 (0.7–2.9)	184	1.1 (0.6–2.0)	204	1.9 (1.0–3.6)	155	1.8 (0.8–3.7)	174	1.8 (0.9–3.5)
*OBC*	500	1.2 (0.5–2.9)	495	1.2 (0.7–2.2)	221	1.5 (0.8–2.6)	433	0.9 (0.5–1.5)	382	2.1 (1.2–3.7)	253	2.5 (1.2–4.9)	326	1.9 (1.0–3.3)
*Forward castes*	106	1	105	1	68	1	99	1	63	1	43	1	61	1
Type of land owned														
*Homestead only*	371	0.4 (0.2–0.7)	356	0.9 (0.5–1.5)	162	1.3 (0.8–1.9)	285	1.5 (1.1–2.1)	288	1.1 (0.8–1.5)	209	1.3 (0.8–2.0)	241	0.9 (0.6–1.3)
*Homestead and other land*	484	1	481	1	240	1	431	1	361	1	242	1	320	1
Household possess MGNREGA job card														
*Yes*	98	0.7 (0.2–1.7)	96	0.9 (0.5–1.5)	50	1.1 (0.6–2.1)	70	1.1 (0.6–1.9)	77	0.9 (0.5–1.5)	54	1.3 (0.7–2.5)	67	1.7 (0.9–3.1)
*No*	757	1	741	1	352	1	646	1	572	1	397	1	494	1
Household possess ration card														
*No*	392	1.1 (0.7–1.9)	384	0.9 (0.6–1.2)	192	0.7 (0.4–1.0)	336	1.1 (0.8–1.5)	295	0.9 (0.7–1.3)	195	0.9 (0.6–1.3)	250	1.1 (0.7–1.5)
*Yes*	463	1	453	1	210	1	380	1	354	1	256	1	311	1
Household’s child feeding affected with government supplementary nutrition program closure														
*Yes*	153	1.7 (0.9–3.1)	151	1.6 (1.1–2.4)	88	1.0 (0.6–1.7)	119	1.4 (0.9–2.1)	120	1.1 (0.7–1.7)	86	0.9 (0.5–1.6)	104	0.8 (0.5–1.4)
*No*	702	1	686	1	314	1	597	1	529	1	365	1	457	1
Took loan /borrow from neighbours/relatives														
*Yes*	560	1.5 (0.8–2.8)	545	1.3 (0.9–1.8)	241	1.5 (0.9–2.2)	452	1.6 (1.1–2.2)	411	1.5 (1.1–2.1)	279	1.3 (0.9–2.0)	357	1.7 (1.1–2.4)
*No*	295	1	292	1	161	1	264	1	238	1	172	1	204	1

*AOR* Adjusted odds ratio, *CI* Confidence interval, *GLVs* Green leafy vegetables, *MPCE* Monthly per capita expenditure, *SC/ST* Schedule castes/schedule tribes, *OBC* Other backward classes, *MGNREGA* Mahatma Gandhi National Rural Employment Guarantee Act

^a^Analysis using logistic regression

Univariate analysis for factors affecting stopping consumption of food items is given in a table (see Additional file 2).

## Discussion

In our longitudinal study sample of 876 predominantly agricultural households in rural Bihar, household diet diversity decreased (diet diversity score fell by one unit) during lockdown compared with pre-COVID-19. Nearly 60% and above reported stopping consumption of nutrient-rich foods such as chicken, fish and eggs although the population was predominantly non-vegetarian. Also, a quarter of the households reporting reduced consumption of staple food items such as cereals (21%) and pulses (25%).

While there are several studies that have reported higher food insecurity due to the pandemic, there are very few that have examined diet diversity and intake of key nutrient-rich foods. Diet diversity is important to support good nutrition, which in turn is important to bolster immunity against infection [28]. This becomes particularly important during the ongoing pandemic. Rural communities particularly those engaged in agriculture are a vulnerable group facing undernutrition [2]. In our study, average household size increased during lockdown possibly because family members working away from home or had migrated were forced to return and/or through child births. This in turn, may have affected household diet diversity probably due to either an increase in number of household members or due to reduced income. In a recent study from Bihar nearly 48% of households reported facing food shortages during the lockdown [29] despite being engaged in agriculture. A study conducted at three time points between May and August 2020 showed a reduction in diet diversity among 833 farmers across 12 states in India [30].

The longitudinal data from our study indicated that the COVID-19 induced lockdown led to disruption in routine food consumption among households, particularly with consumption of nutrient-rich foods such as animal source foods and fruits. Other studies across various states in India have also shown similar results. For example, a survey conducted in May 2020 with 448 participants from 4 States, 62% reported disruption in their routine food consumption with reduced access to mainly nutrient-rich foods such as animal-sourced foods and fruits [31]. In our study, non-agricultural households with labourers mainly belonged to low social class (39%) and were likely dependent on food purchase for their food intake. A systematic review on the impact of COVID-19 on nutritional status of populations in LMICs has raised concerns on the long term impacts on access to and affordability of nutrient-rich, healthy diets and their health implications, particularly among women and individuals belonging to low socio-economic groups [32]. Decreased food consumption may have been due to several collateral impacts of the pandemic such as reduced income that affect food security [32].

Nutrient-rich foods are generally more expensive than cereals, and the lockdown caused further spike in prices of most nutrient-rich foods [33, 34], further affecting affordability of the already limited purchasing power of rural communities [35]. Evidence from over 30,000 households in 16 original household surveys from nine countries in Africa (Burkina Faso, Ghana, Kenya, Rwanda, Sierra Leone), Asia (Bangladesh, Nepal, Philippines), and Latin America (Colombia) found a 67% median income decline causing widespread food insecurity in these regions [36]. In our analysis, although household expenditure was not associated with low diet diversity, households with a financial burden of loan from neighbours/relatives had higher odds of low diet diversity. Households that were forced to cut food intake were also probably forced to take loans to make ends meet, but the amounts were not enough to buffer consumption levels. These households may have prioritized the use of the money for other needs. The household having ‘taken loan’ could be considered as an indication of a household financial crisis, and such households were likely to have reduced diet diversity. Another analysis on this data [19] has shown that households which received small cash transfers (~ 6.67 USD per month) by the government during the lockdown [18] had lower odds of being food insecure which was assessed using the food insecurity experience scale [37]. However, the findings from this study show that such small cash transfers were insufficient to provide adequate diet diversity.

Our study findings need to be interpreted considering some limitations. While we cannot directly state that the findings of this study were attributable to the COVID-19 induced lockdown, the underlying mechanisms through which the lockdown could affect food consumption, and the consistency of the effect of the lockdown across various states in India, are supportive. Self-reported data may have been influenced by a social desirability bias. With data collected telephonically, we were unable to collect quantitative data of food consumed to substantiate our results. Nevertheless, the strength of our study lies in the analysis of the longitudinal data with an available pre-COVID-19 household food consumption baseline.

In anticipation of future disruptions due to further lockdowns that can affect food consumption among vulnerable populations, certain measures need to be planned well in advance to mitigate potential effects. Direct cash transfers to vulnerable sections of society may offer individuals flexibility in spending for their household needs and food consumption [38]. The PM Kisan scheme [39], a direct cash transfer to landholding farmers, providing INR 6000 (approximately 79 USD) per year in 3 equal installments, could be provided in an aggregate lump-sum in the sowing season to ease economic constraints. In the short-term, distribution of nutrient-rich foods, in addition to the presently distributed staples, among impoverished societal sections through existing government schemes such as the National Food Security Act, 2013 and the PDS can be advocated. The sustenance of government supplementary nutrition programs to children, such as the Integrated Child Development Scheme and school midday meal program, even during lockdown, must be planned. In the long term, building resilient food systems by promoting farming with a focus on nutrition can help improve household diet diversity and strengthen local value chains [40], particularly in case of nutrient-rich foods. The COVID-19 pandemic should be seen as a wake-up call to build this long-term local food system resilience to maintain adequate food consumption in rural communities.

## Conclusions

This study contributes to the understanding of the impact of COVID-19 on diet diversity and food consumption among low-income rural households in India. COVID-19 has impacted the consumption of nutrient-rich foods in these households. Maintaining diet diversity among socio-economically vulnerable households during periods when food consumption is most threatened by shocks i.e. COVID-19 would need sustained government support in terms of social protection coverage and benefit transfers in rural communities.

## Supplementary Information

**Additional file 1.** Univariate analysis for factors affecting reduction in consumption of food items ^d^.

**Additional file 2.** Univariate analysis for factors affecting stopping consumption of food items ^d^.

**Additional file 3.** STROBE Statement—Checklist of items that should be included in reports of cohort studies.

## Data Availability

The datasets used and/or analysed during the current study are available from the corresponding author on reasonable request.

## Abbreviations

AOR: Adjusted odds ratio
CI: Confidence interval
COVID-19: Coronavirus disease
GLVs: Green leafy vegetables
HDDS: household diet diversity score
LMICs: Low- and middle-income countries
MGNREGA: Mahatma Gandhi National Rural Employment Guarantee Act 2005
MPCE: Monthly per capita consumer expenditure
OBC: Other Backward Classes
OR: Odds ratio
PDS: Public distribution system
SC/ST: Scheduled Castes/Scheduled Tribes
SPSS: Statistical Package for the Social Sciences
USD: United States Dollar
