# Supplementary material for: Impact of the COVID-19 lockdown on household diet diversity in rural Bihar, India: a longitudinal survey
Source: Nutr J. 2023 Feb 27;22:13. doi: 10.1186/s12937-023-00842-z (PMC9968637; doi:10.1186/s12937-023-00842-z)
Supplement: Supplementary file 1 — Additional file 1. Univariate analysis for factors affecting reduction in consumption of food items d. [file 12937_2023_842_MOESM1_ESM.pdf]

Additional file 1: Univariate analysis for factors affecting reduction in consumption of food items [d]

	Cereals		Pulses		GLVs		Fruits		Milk		Egg		Fish		Chicken	
	N	OR (95%CI)	N	OR (95%CI)	N	OR (95%CI)	N	OR (95%CI)	N	OR (95%CI)	N	OR (95%CI)	N	OR (95%CI)	N	OR (95%CI)
**MPCE**																
<=1247.4	168	1 (0.6-1.7)	153	1.1 (0.6-1.8)	124	1.1 (0.6-1.9)	34	1.3 (0.5-3.1)	78	2 (1.0-3.9)	53	1.2 (0.5-2.5)	24	3 (1.0-9.0)	32	3.7 (1.3-10.1)
1247.5 - 1674.5	174	1.5 (0.9-2.6)	158	1.6 (0.9-2.6)	120	0.7 (0.4-1.4)	37	0.6 (0.2-1.6)	83	1.4 (0.7-2.8)	51	0.9 (0.4-2.0)	39	1.3 (0.4-3.4)	42	2.3 (0.9-6.1)
1674.6 - 2150.1	173	1.6 (0.9-2.7)	158	1.3 (0.8-2.2)	139	1 (0.6-1.8)	48	1.2 (0.5-2.8)	100	1.1 (0.5-2.1)	57	1.2 (0.5-2.5)	27	2 (0.7-5.9)	40	1.5 (0.5-4.1)
2150.2 - 2947.0	171	0.9 (0.5-1.6)	160	1.1 (0.6-1.8)	125	1.1 (0.6-2.0)	44	1.2 (0.5-2.8)	96	1.2 (0.6-2.3)	53	1.4 (0.7-3.0)	41	3.1 (1.2-8.1)	44	1.1 (0.4-2.9)
>2947.0	174	1	159	1	135	1		1	115	1	62	1	36	1	39	1
**Social group**																
SC/ST	251	3.6 (1.7-7.3)	230	1.8 (1.0-3.3)	178	2.3 (1.2-4.6)	56	1.5 (0.6-3.5)	110	3.6 (1.6-8.1)	84	1.5 (0.6-3.4)	58	1.9 (0.7-5.3)	58	0.8 (0.3-2.1)
OBC	503	2.5 (1.2-5.0)	459	1.7 (1.0-3.0)	379	1.9 (1.0-3.7)	114	1.5 (0.7-3.1)	293	2.2 (1.0-4.6)	154	1.6 (0.7-3.4)	84	1.4 (0.5-3.7)	109	0.7 (0.3-1.5)
Forward castes	106	1	99	1	86	1	42	1	69	1	38	1	25	1	30	1
**Type of land owned**																
Homestead only	374	0.7 (0.5-1.1)	353	1.4 (1.0-2.0)	271	1.9 (1.2-2.7)	78	2.3 (1.3-4.0)	167	1.8 (1.1-2.7)	116	1.3 (0.8-2.2)	71	1.2 (0.6-2.2)	85	1.1 (0.6-2.0)
Homestead and other land	486	1	435	1	372	1	134	1	305	1	160	1	96	1	112	1
**Household possess MGNREGA job card**																
Yes	98	1.4 (0.9-2.3)	92	1.3 (0.8-2.1)	73	1.3 (0.8-2.3)	23	4.1 (1.5-11.0)	43	1.2 (0.6-2.5)	32	2 (0.9-4.3)	16	4.7 (1.4-15.3)	16	5.8 (1.8-18.9)
No	762	1	696	1	570	1	189	1	429	1	244	1	151	1	181	1
**Household possess ration card**																
No	395	0.8 (0.6-1.1)	359	0.9 (0.7-1.3)	300	0.8 (0.5-1.2)	110	0.8 (0.4-1.4)	220	1 (0.6-1.5)	127	0.7 (0.4-1.2)	76	0.3 (0.2-0.7)	87	0.5 (0.2-0.9)
Yes	465	1	429	1	343	1	102	1	252	1	149	1	91	1	110	1
**Household's child feeding affected with government supplementary nutrition program closure**																
Yes	155	1.6 (1.0-2.3)	136	1.1 (0.7-1.7)	105	1.7 (1.1-2.7)	44	2.6 (1.3-5.2)	70	1.6 (0.9-2.8)	47	1.2 (0.6-2.3)	30	2 (0.9-4.5)	38	1.2 (0.6-2.6)
No	703	1	652	1	538	1	168	1	402	1	229	1	137	1	159	1
**Took loan from neighbours/relatives**																
Yes	562	1.7 (1.1-2.4)	510	2.5 (1.7-3.6)	408	1.5 (1.0-2.2)	117	1.5 (0.8-2.6)	280	1.7 (1.1-2.7)	158	1.4 (0.9-2.4)	97	1.6 (0.8-3.1)	109	1.7 (0.9-3.1)

No	298	1	278	1	235	1	95	1	192	1	118	1	70	1	88	1

OR: odds ratio; CI: confidence interval; GLVs: Green leafy vegetables; MPCE: monthly per capita expenditure; SC/ST: schedule castes/schedule tribes; OBC: other backward classes; MGNREGA: Mahatma Gandhi National Rural Employment Guarantee Act

[d] Analysis using logistic regression
