# Supplementary material for: Impact of the COVID-19 lockdown on household diet diversity in rural Bihar, India: a longitudinal survey
Source: Nutr J. 2023 Feb 27;22:13. doi: 10.1186/s12937-023-00842-z (PMC9968637; doi:10.1186/s12937-023-00842-z)
Supplement: Supplementary file 2 — Additional file 2. Univariate analysis for factors affecting stopping consumption of food items d. [file 12937_2023_842_MOESM2_ESM.pdf]

Additional file 2: Univariate analysis for factors affecting stopping consumption of food items [d]

	Pulses		GLVs		Fruits		Milk		Egg		Fish		Chicken	
	N	OR (95%CI)	N	OR (95%CI)	N	OR (95%CI)	N	OR (95%CI)	N	OR (95%CI)	N	OR (95%CI)	N	OR (95%CI)
**MPCE**														
<=1247.4	165	0.7 (0.3-1.7)	166	1.4 (0.8-2.3)	73	1.3 (0.7-2.5)	129	1.8 (1.1-2.9)	125	1.2 (0.7-2.0)	79	1.5 (0.8-2.8)	110	1.3 (0.7-2.3)
1247.5 - 1674.5	173	1.0 (0.4-2.1)	168	1.5 (0.9-2.6)	72	1.0 (0.5-1.9)	139	1.7 (1.1-2.9)	134	1.3 (0.8-2.2)	99	0.9 (0.5-1.7)	113	0.8 (0.5-1.5)
1674.6 - 2150.1	173	1.0 (0.5-2.2)	170	0.8 (0.5-1.5)	82	0.8 (0.4-1.4)	141	1.0 (0.6-1.7)	132	1.1 (0.7-1.8)	90	1.4 (0.7-2.6)	111	0.9 (0.5-1.6)
2150.2 - 2947.0	171	0.7 (0.3-1.5)	164	1.2 (0.7-2.1)	81	0.9 (0.5-1.6)	149	1.3 (0.8-2.2)	123	1.0 (0.6-1.7)	89	0.6 (0.3-1.2)	115	0.8 (0.4-1.3)
>2947.0	173	1	169	1	94	1	158	1	135	1	94	1	112	1
**Social group**														
SC/ST	249	1.1 (0.4-2.8)	237	1.5 (0.8-2.6)	113	1.6 (0.8-3.0)	184	1.5 (0.9-2.6)	204	2.1 (1.2-3.8)	155	2.3 (1.1-4.6)	174	1.9 (1.0-3.5)
OBC	500	1.2 (0.5-2.8)	495	1.3 (0.8-2.3)	221	1.5 (0.8-2.6)	433	1.0 (0.6-1.7)	382	2.2 (1.3-3.8)	253	2.7 (1.4-5.4)	326	1.9 (1.1-3.3)
Forward castes	106	1	105	1	68	1	99	1	63	1	43	1	61	1
**Type of land owned**														
Homestead only	371	0.4 (0.2-0.8)	356	1.0 (0.7-1.4)	162	1.4 (0.9-2.1)	285	1.7 (1.2-2.3)	288	1.2 (0.9-1.6)	209	1.3 (0.8-1.9)	241	1.0 (0.7-1.4)
Homestead and other land	484	1	481	1	240	1	431	1	361	1	242	1	320	1
**Household possess MGNREGA job card**														
Yes	98	0.7 (0.3-1.7)	96	1.0 (0.6-1.7)	50	1.3 (0.7-2.4)	70	1.2 (0.7-2.0)	77	1.0 (0.6-1.6)	54	1.4 (0.7-2.6)	67	1.8 (1.0-3.3)
No	757	1	741	1	352	1	646	1	572	1	397	1	494	1
**Household possess ration card**														
No	392	1.0 (0.6-1.7)	384	0.8 (0.6-1.2)	192	0.7 (0.4-1.0)	336	1.0 (0.7-1.4)	295	0.9 (0.7-1.3)	195	0.8 (0.5-1.2)	250	1.0 (0.7-1.4)
Yes	463	1	453	1	210	1	380	1	354	1	256	1	311	1

**Household’s child feeding affected with government supplementary nutrition program closure**														
Yes	153	1.6 (0.9-2.9)	151	1.5 (1.0-2.5)	88	1.0 (0.6-1.7)	119	1.4 (0.9-2.1)	120	1.1 (0.7-1.7)	86	1.1 (0.7-1.8)	104	0.9 (0.6-1.4)
No	702	1	686	1	314	1	597	1	529	1	365	1	457	1
**Took loan from neighbours/relatives**														
Yes	560	1.6 (0.9-2.9)	545	1.3 (0.9-1.9)	241	1.5 (1.0-2.3)	452	1.7 (1.2-2.4)	411	1.6 (1.1-2.2)	279	1.3 (0.9-1.9)	357	1.7 (1.2-2.5)
No	295	1	292	1	161	1	264	1	238	1	172	1	204	1

OR: odds ratio; CI: confidence interval; GLVs: Green leafy vegetables; MPCE: monthly per capita expenditure; SC/ST: schedule castes/schedule tribes; OBC: other backward classes; MGNREGA: Mahatma Gandhi National Rural Employment Guarantee Act

[d] Analysis using logistic regression
